# Study of water-conducting fractured zone development law and assessment method in longwall mining of shallow coal seam

**DOI:** 10.1038/s41598-022-12023-9

**Published:** 2022-05-14

**Authors:** Xiaobin Li, Dongliang Ji, Penghua Han, Quansheng Li, Hongbao Zhao, Fulian He

**Affiliations:** 1 School of Energy and Mining Engineering, China University of Mining and Technology-Beijing, Beijing, 100083 China; 2 State Key Laboratory of Water Resource Protection and Utilization in Coal Mining, Beijing, 102209 China

**Keywords:** Natural hazards, Civil engineering

## Abstract

Starting from the source of mining, scientific understanding of surface damage law and assessment method in longwall mining of shallow coal seam is conducive to solving the problems of geological hazards and deterioration of the ecological environment, and promoting the coordinated development of efficient coal mining and environmental protection. Based on numerical simulation and theoretical analysis, the surface damage process and spatiotemporal evolution of fracture field are discussed. The influencing factors and assessment method of surface damage are clarified. The results show that surface damage undergone the immediate roof caving stage, the fracture and instability stage of main roof, the spatial amplification stage of separation layer, the instability stage of surface damage control layer and the mining damage stability stage. Under the critical extraction condition, the cracks above the goaf are divided into the crack area outside the cut, the crack area inside the cut, the re-compaction area in the middle goaf, the crack area behind the longwall face, and the crack area in front of the longwall face. The overburden reaches critical failure ahead of surface critical mining. The sensitivity of loose layer thickness to surface subsidence coefficient is greater than that of mining thickness to surface subsidence coefficient. Surface damage control should be adjusted to local conditions, and finally realize zoning treatment and zoning repair. Through the three-step method of "longwall face rapid advancing method, local grouting reinforcement overburden method and zoning treatment ground fissures method", the surface damage control of 12,401 longwall face is realized. This research provides theoretical guidance and application value for surface ecological restoration in similar mining area.

## Introduction

Shendong mining area is a typical representative of high-intensity mining in Western mining area. The longwall mining is characterized by shallow buried depth, large mining height, fast advancing speed, high mechanization of mining equipment, serious overburden damage and surface damage, resulting in a large number of overburden fractures and ground fissures^1–3^. High-intensity mining causes dramatic changes in the groundwater resource system, affecting the surface soil moisture, organic matter and mineral content. In addition, the fragile ecological environment in the western region has resulted in the increasingly prominent contradiction between efficient coal mining and ecological protection^4–6^. Therefore, it is urgent to study the law of surface damage and control technology from the source of mining.

Mining induced damage refers to the process of changing the stress state of surrounding rock after coal mining, forming high and low stress areas, resulting in deformation, damage and migration of overburden, and finally transmitted to the surface, leading to mining subsidence and ecological damage^7,8^. The essence of mining damage is the law of the influence of rock movement on safety and environment, and it is also an important scientific problem of green coal mining^9,10^. Some mining experts and scholars have also carried out a series of research on mining damage, using key strata, elastic thin plate, arch structure, "hyperbolic like" model and other theories to reveal the influence mechanism of strata movement on mining subsidence and ground fissures^11–14^, and the internal relationship between roof structure (asymmetric triple hinged arch, short masonry beam, stepped rock beam) and mine pressure behavior^15–17^. The above research promotes the further development of strata movement theory, and is conducive to guiding the field practice scientifically.

Many scholars have also discussed the height of overburden failure. Majdi et al.^18^ clarified five mathematical method to determine the height of fracture zone and caving zone. Based on the rock failure criteria, Guo et al.^19^ developed a new method to predict the height of fracture water-conducting zone and successfully applied to Tongxin coal mine. Li et al.^20^ studied the overburden failure law of extremely close seam by physical similarity simulation. Xu et al.^21^ established a trapezoidal mechanical model to explain and control water inrush induced by longwall mining. Li et al.^22^ proposed a mathematical evaluation model to determine the influencing factors sensitivity of fracture water-conducting zone. Zhang et al.^23^ analysed the failure range of overburden by geophysical exploration method. These previous works have provided innovative theoretical support for overburden failure under different geological conditions.

In terms of surface subsidence, it mainly includes subsidence prediction and subsidence area treatment. Cao et al.^24^ established a mechanical model to predict surface subsidence and successfully applied to Ningtiaota coal mine. Xu et al.^25^ and Zhu et al.^26^ obtained that the stability of key stratum is the main factor affecting the surface subsidence. Meanwhile, Wu et al.^27^ found the internal relationship between hard and thick rock strata and surface subsidence. Hou et al.^28^ developed that the softer the rock stratum, the greater the surface subsidence. Karmis et al.^29^ adopted mathematics function method to calculate maximum subsidence. Singh et al.^30^ proposed a visco and elastic model to determine surface subsidence value in Indian mining area. In addition, Zhu et al.^31^ developed the backfill-strip mining to reduce surface subsidence and successfully applied Ezhuang coal mine. Wang et al.^32^ used the theory of fuzzy matter-element and the combined weight to control subsidence in thick loose layer. Xuan et al.^33^ adopted grouting injection technology from surface boreholes to control subsidence. Li et al.^34^ proposed the mining source loss technology based on parameter optimization. Besides, other mining methods (thickness limit mining, strip mining, filling mining, mining-filling-retaining coordination mining), retaining coal pillars, overburden separation grouting technology are also adopted to control subsidence^35–38^.

As described above, experts mainly focuses on the height of overburden failure, prediction and control of surface subsidence. However, studies regarding the process and influencing factors of surface damage are rare. In addition, quantifying the damage range of overburden and appropriate longwall face advance still remains unclear. Surface damage control does not comprehensively consider the longwall face, overburden and surface. Therefore, based on typical longwall face 12,401 in Shangwan coal mine, combining with numerical simulation, theoretical analysis, and field application, the paper obtains five stages of surface damage in high-intensity mining, the corresponding longwall face advance of the overburden critical failure and surface critical mining. The assessment method of surface damage is established. Three-step control method of "longwall face rapid advancing method, local grouting reinforcement overburden method and zoning treatment ground fissures method" is proposed. This research can reduce the degree of surface damage and achieve the goal of protecting the ecological environment.

## Project background

### Mining and geological condition

**Figure 1:**
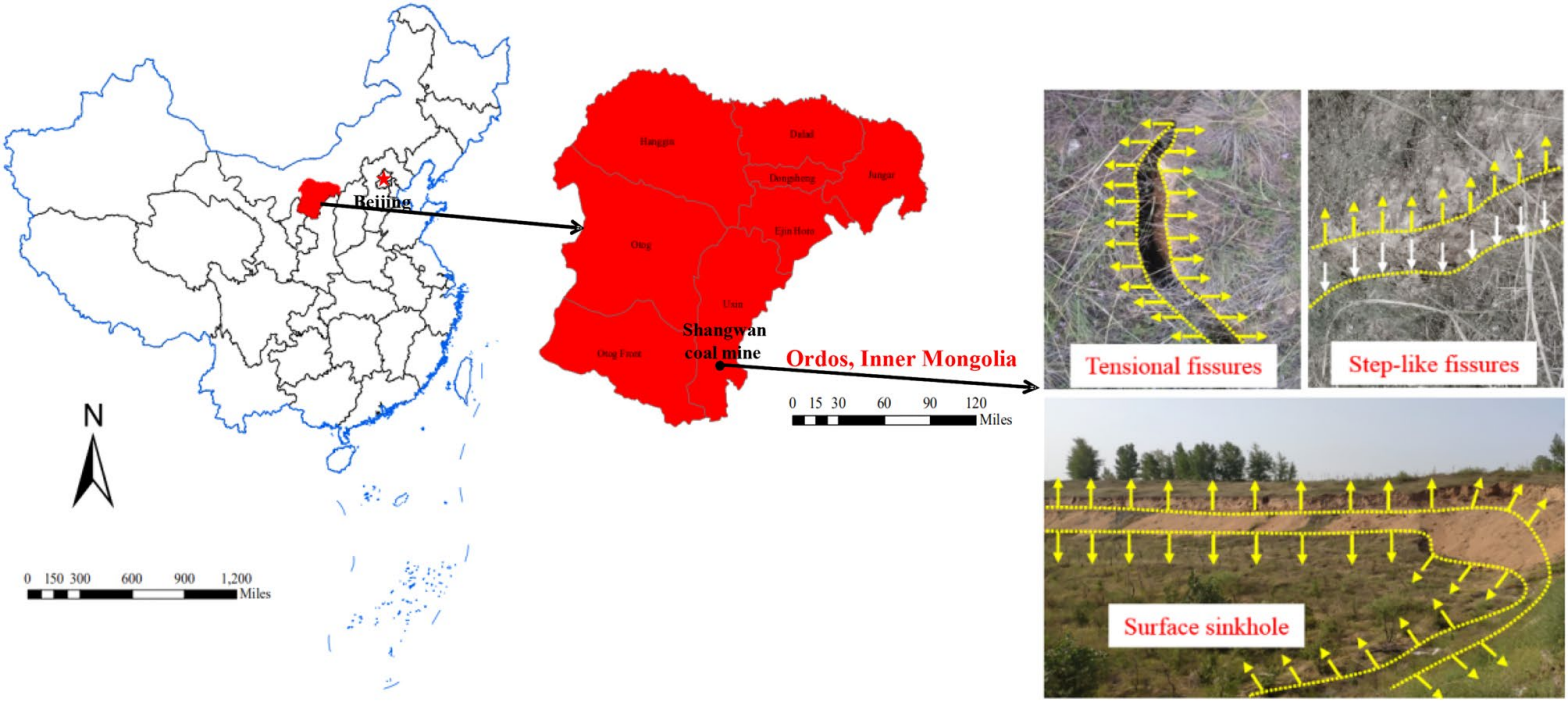
Distribution of ground fissures.

Taking the 12,401 longwall face of Shangwan coal mine as the engineering background, the average buried depth is 220 m, the mining height is 8.8 m, the length of longwall face is 300 m, and the average advancing speed is 12 m/d, which belongs to the high-intensity longwall mining. The thickness of the overlying bedrock is 120–224 m, and the thickness of the overlying loose layer is 0–27 m, which is mainly yellowish medium and fine-grained aeolian sand, loose and unconsolidated. Due to the high-intensity mining, the surface damage control layer is broken, and a large number of ground fissures and collapse troughs are generated on the surface. The surface damage distribution is shown in Fig. 1. Through on-site investigation, it was found that the types of ground fissures include surface sinkhole, tensional fissures and step-like fissures.

### Overview of the methodology

**Figure 2:**
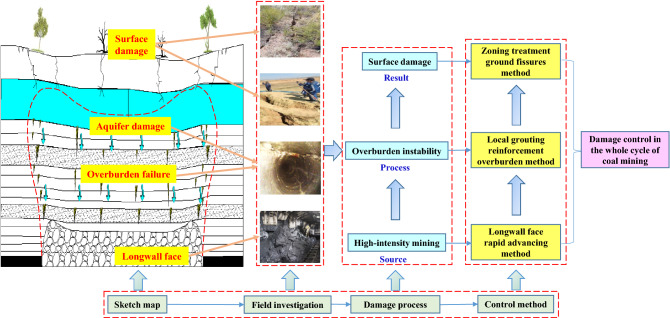
Overview of the methodology.

Through field investigation, numerical simulation and theoretical analysis, this study analyzes the surface damage process of longwall mining, and finally has been applied in the field. An overview of the methodology in this study is shown in Fig. 2.

## Numerical simulation

UDEC 7.0 (https://www.itascacg.com/software/UDEC) is a two-dimensional numerical calculation software based on discontinuous discrete element method. It mainly simulates the mechanical behavior of discontinuous media (such as jointed blocks) under static or dynamic load. Discontinuous media is reflected by the combination of discrete blocks. Joints are treated as boundary conditions between blocks, allowing blocks to move and rotate along the joint surface. At present, UDEC 7.0 is an ideal software to simulate the movement of rock stratum and surface subsidence under different lithology and mining conditions. It can better simulate the process of roof separation, caving and re-compaction after coal seam mining, and can accurately analyze the breaking law of overburden^39^. The constitutive model used in this simulation is Mohr–Coulomb model.

**Figure 3:**
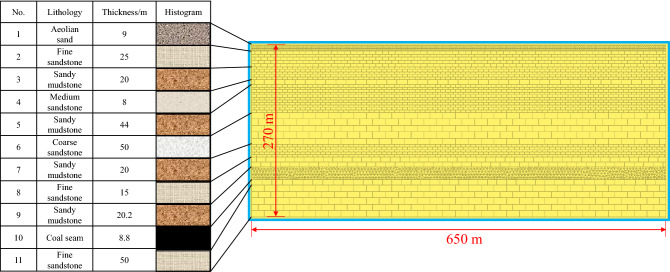
Numerical model.

**Figure 4:**
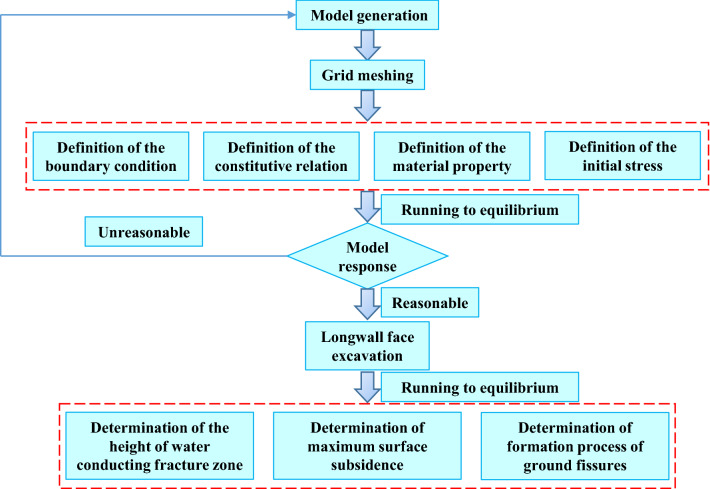
The flow chart of modeling process.

**Figure 5:**
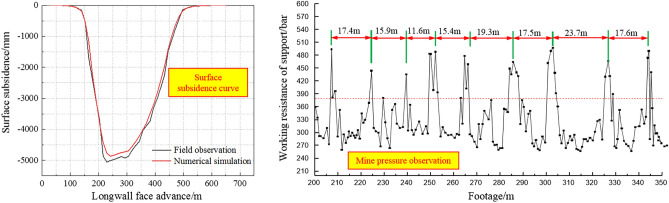
The validation of numerical model.

In order to facilitate the establishment of numerical model and grid division, the thickness of some rock strata is rounded and combined. Numerical model and the flow chart of modeling process is shown in Figs. 3 and 4. The model size is 650 m (length) × 270 m (high). Considering the influence of boundary coal pillar, 160 m is reserved on both sides. Step by step excavation method is adopted, with each excavation length of 15 m. The total excavation length of longwall face is 330 m. The physical and mechanical parameters of coal and rock strata are shown in Table 1. As shown in Fig. 5, mine pressure observation shows that the average periodic weighting step of the main roof is 15 m, which is consistent with the numerical simulation results. Meanwhile, the maximum surface subsidence of field observation is 5055 mm, while the maximum surface subsidence of numerical simulation is 4923 mm. The above results verify the reliability of numerical model.

**Table 1:** Physical and mechanical parameters of coal and rock strata.

Lithology	Density/(kg m^−3^)	Bulk modulus/GPa	Shear modulus/GPa	Cohesion/MPa	Friction angle/(°)	Tensile strength/MPa
Aeolian sand	1580	3.3	1.5	–	–	–
Fine sandstone	2500	8.3	4.3	4.1	40	2.9
Sandy mudstone	2400	2.3	1.2	5.9	37	3.0
Medium sandstone	2390	8.0	4.8	4.7	41	3.7
Coarse sandstone	2430	7.6	3.9	6.0	43	4.9
Coal seam	1480	1.7	0.6	2.8	40	0.7

### Ground fissures formation process

**Figure 6:**
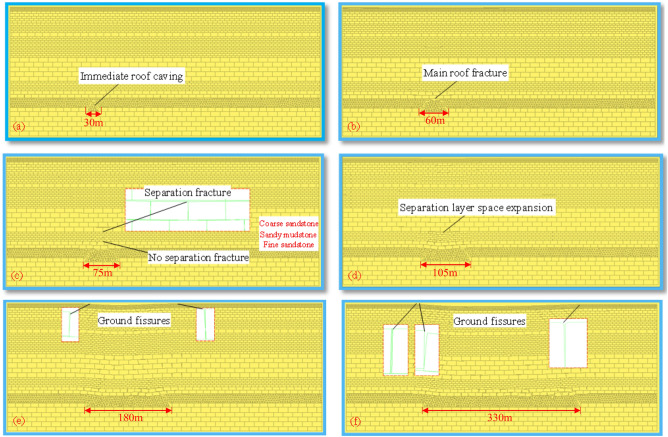
Ground fissures formation process. (**a**) 30 m advancement. (**b**) 60 m advancement. (**c**) 75 m advancement. (**d**) 105 m advancement. (**e**) 180 m advancement. (**f**) 330 m advancement.

Ground fissures formation process is shown in Fig. 6. When the longwall face advances 30 m, the immediate roof collapses; When the longwall face advances 60 m, the main roof occurs the primary fracture; When the longwall face advances 75 m, separation crack appears at the interface of coarse sandstone and sandy mudstone, and there is no separation crack at the interface of sandy mudstone and fine sandstone. This is due to the different mechanical properties of each rock layer, resulting in asynchronous bending and subsidence. Generally, the separation cracks are more likely to occur at the combination interface of upper hard rock and lower soft rock. When the longwall face advances 105 m, the separation layer space is further expanded, and the surface slightly sinks. When the longwall face advances 180 m, V-shaped tensile ground fissures appear on the surface; When the longwall face advances 330 m, the surface reaches critical mining, the excavation length is 1.5 times of the buried depth, and a series of discontinuous deformation occurs. It can be seen from the above that the formation process of ground fissures is divided into the stage of overburden breakage and migration and the stage of surface soil deformation. With the advancement of the longwall face, the ground fissures undergone three stages of "generation-expansion-closing", and the overall distribution is in a "V" shape. The closure of ground fissures are caused by the rotation movement of overburden, indicating that the crack has the phenomenon of self-repair. This research lays a solid foundation for the development of ground fissures treatment technology.

**Figure 7:**
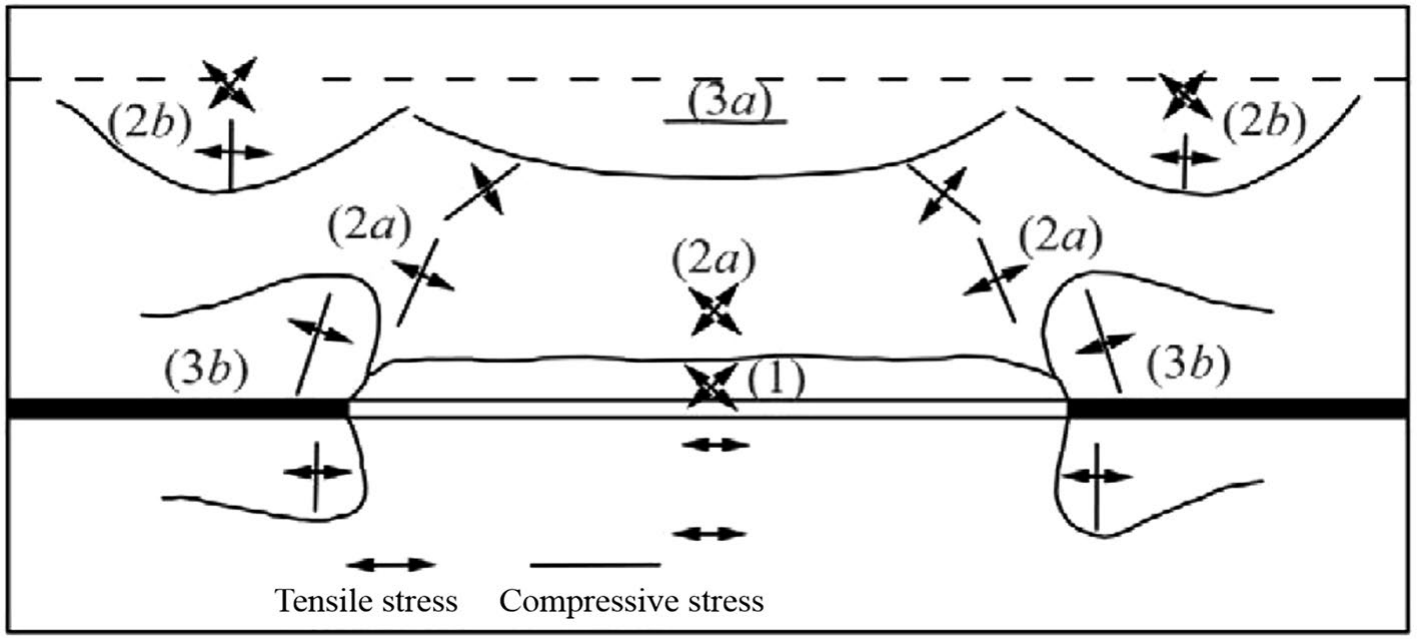
The stress redistribution around longwall face^42^. 1—Tensile stress zone above the goaf; 2a—Tensile and compressive stress zone above the goaf; 2b—Tensile and compressive stress zone at the top; 3a—Compressive stress zone at the top; 3b—Abutment pressure zone.

After the longwall face is mined, the initial stress equilibrium state is destroyed, causing inhomogeneous subsidence and moving deformation at various points on the surface. When the stress on the topsoil exceeds its ultimate strength and the discontinuous deformation exceeds the ultimate deformation of the topsoil, the soil is damaged along the original cracks to form ground fissures^40,41^. The ultimate strength is different due to different stress modes, among which the tensile stress is the most likely to damage the rock and soil. Based on the reference^42^, the stress redistribution around longwall face is shown in Fig. 7.

### Development law of mining crack field

**Figure 8:**
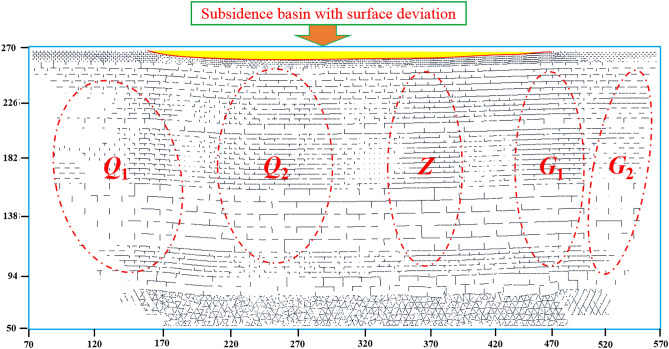
Development law of mining crack field. (***Q***_**1**_) The crack area outside the open-off cut. (***Q***_**2**_) The crack area inside the open-off cut. (***Z***) The re-compaction area in the middle goaf. (***G***_**1**_) The crack area behind the longwall face. (***G***_**2**_) The crack area in front of the longwall face.

The development law of mining crack field is shown in Fig. 8. Initially the mining cracks are mainly distributed in the collapsed immediate roof, and the fracture of the main roof further expands the cracks range. The density of the cracks is related to the lithology of rock strata. Some primary cracks are closed due to the compaction of the collapsed strata in the goaf, and new cracks are generated above the goaf. In the vertical direction, there are no cracks in the middle, but there are cracks in the upper and lower layers. This is due to the uneven subsidence of the surface caused by high-intensity mining, resulting in downward cracks. In the process of mining damage from the coal seam to the surface, the mining damage energy is gradually decreasing, and the development ends in a certain rock layer. At this time, there is no mining crack area between the upward cracks and the downward cracks. With the advancement of longwall face, the cracks undergone the generation, expansion, and compaction. After the surface reaches critical mining, the crack area outside the open-off cut, the crack area inside the open-off cut, the re-compaction area in the middle goaf, the crack area behind the longwall face, and the crack area in front of the longwall face are generated above the goaf, forming a subsidence basin with surface deviation. There are mainly closed cracks in the middle of goaf and shear cracks on both sides of longwall face. The main channel of water and sand is between *Q*_1_ and *Q*_2_, *G*_1_ and *G*_2_. From the horizontal relative damage ratio greater than 1, it can be seen that the mining damage range of the open-off cut side is greater than that of the longwall face side, and the maximum subsidence of the surface is inclined to the open-off cut side, which is related to the asymmetric rock fracture angle, and the deviation collapse of the overburden is transmitted to the surface, resulting in the deviation subsidence of the surface.

**Figure 9:**
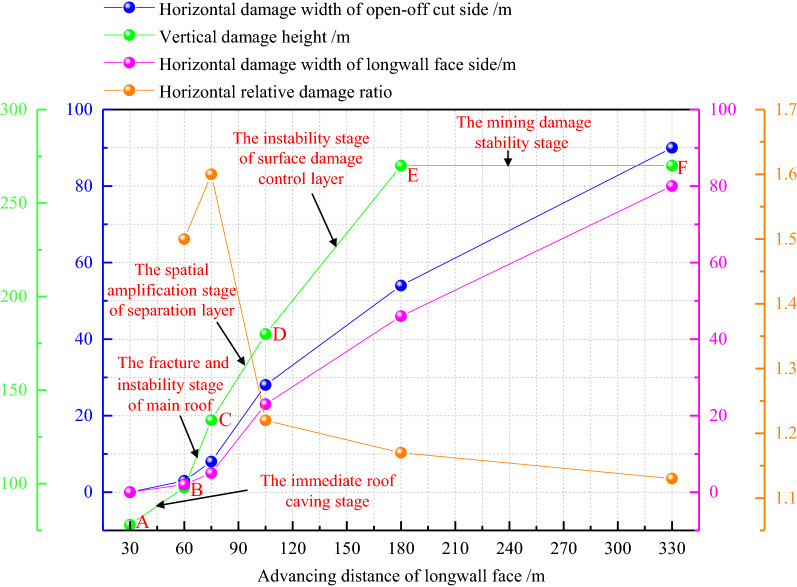
Damage development curve.

It can be seen from Fig. 9 that the damage of high-intensity mining successively undergone the immediate roof caving stage (AB section), the fracture and instability stage of main roof (BC section), the spatial amplification stage of separation layer (CD section), the instability stage of surface damage control layer (DE section) and the mining damage stability stage (EF section). In BC section, CD section and DE section, the damage rate is faster, which indicates that the failure of the main roof and the spatial expansion of the separation layer cause the fracture of the surface damage control layer. When the longwall face advances 180 m, the vertical damage height reaches the maximum of 220 m and remains unchanged. The horizontal damage width of open-off cut and longwall face side are increasing, and the horizontal damage range is increasing all the time. In this paper, the ratio of the horizontal damage width of open-off cut and longwall face side is defined as the horizontal relative damage ratio. The horizontal relative damage ratio first increases (1.50–1.60) and then decreases (1.60–1.13), and the values are greater than 1. The results show that the overall damage range of the open-off cut side is larger than that of the longwall face side, but the increase extent of the open-off cut side first increases and then decreases. The reason is that with the increase of the goaf size, the horizontal relative damage ratio caused by the main roof fracture increases, and then collapsed strata in the goaf gradually tend to be stable. The supporting effect on the overlying strata is transformed from the initial coal wall support to the joint support of the coal wall and the collapsed strata, resulting in the horizontal relative damage ratio gradually decreasing.

## Influencing factors of high-intensity mining damage

After high-intensity coal mining, the overlying strata gradually bend and fracture from the immediate roof, and the damage development from the longwall face to the surface, causing a series of continuous and discontinuous deformation on the surface, threatening the fragile ecological environment in Western China. It is of great significance to clarify the influencing factors of mining damage for the realization of surface restoration and the protection of ecological environment.

### Mining damage simulation scheme

**Figure 10:**
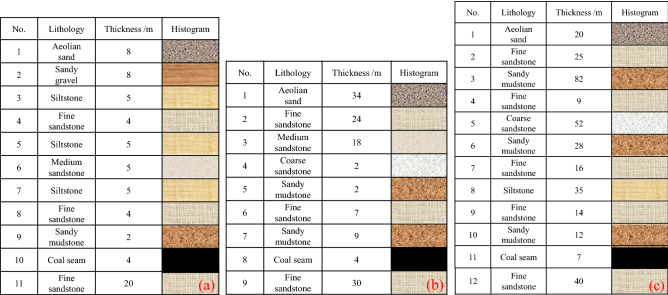
Distribution of coal and rock strata with different burial depth. (**a**) Buried depth 50 m. (**b**) Buried depth 100 m. (**c**) Buried depth 300 m.

Based on the buried depth and mining thickness of longwall face in Shendong mining area, the coal seams involved are all near horizontal. The scheme design is shown in Table 2, and the distribution of coal and rock strata with different buried depths is shown in Fig. 10. In this study, the 12,401 longwall face of Shangwan coal mine is taken as the reference to make the surface subsidence coefficient consistent with the field measured results, and then other simulation schemes adopt its physical and mechanical parameters on this basis.

**Table 2:** Scheme design.

Buried depth *H*/m	Mining thickness *M*/m	Longwall face advance *D*/m	Height of waterconducting fractured zone *H*_1_/m	Surface subsidence *W*/mm	*D*/*H*	*H*/*M*	*W*/*M* (subsidence coefficient)	*H*_1_/*H*
50	4	80	50	1442	1.60	12.5	0.36	1
	4	70	50	1434	1.40	12.5	0.36	1
	4	50	50	295	1.00	12.5	0.07	1
	4	40	45	202	0.80	12.5	0.05	0.9
	4	30	30	140	0.60	12.5	0.04	0.6
100	4	160	100	1890	1.60	25	0.47	1
	4	140	100	1887	1.40	25	0.47	1
	4	100	100	1056	1.00	25	0.26	1
	4	90	66	632	0.90	25	0.16	0.66
	4	80	42	401	0.80	25	0.10	0.42
220	8.8	300	114	4923	1.36	25	0.56	0.52
	8.8	270	114	4900	1.23	25	0.56	0.52
	8.8	180	114	3819	0.82	25	0.43	0.52
	8.8	150	84	2573	0.68	25	0.29	0.38
	8.8	120	64	1016	0.55	25	0.12	0.29
300	7	390	110	4930	1.30	43	0.70	0.37
	7	375	110	4878	1.25	43	0.70	0.37
	7	270	110	3761	0.90	43	0.54	0.37
	7	240	95	3417	0.80	43	0.49	0.32
	7	210	87	2886	0.70	43	0.41	0.29

### Result analysis

**Figure 11:**
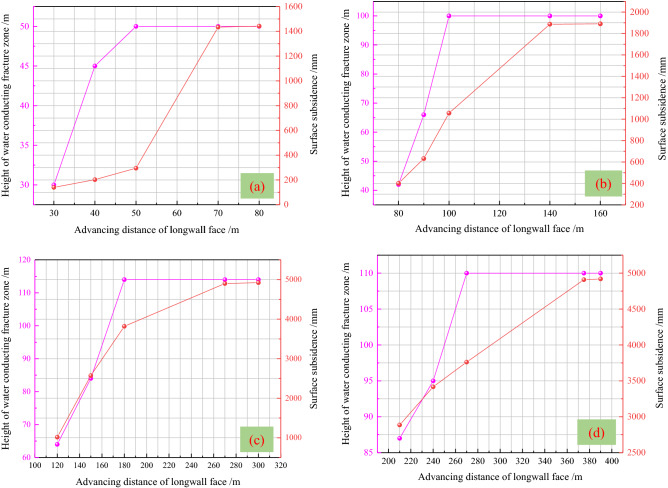
Variation law of water-conducting fractured zone and surface subsidence. (**a**) Buried depth 50 m. (**b**) Buried depth 100 m. (**c**) Buried depth 220 m. (**d**) Buried depth 300 m.

**Figure 12:**
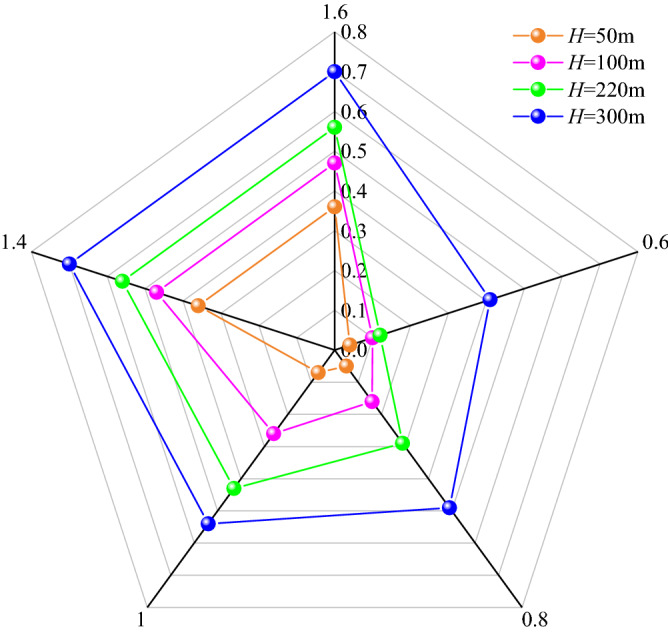
Relationship between *D*/*H* and *W*/*M*.

It can be seen from Figs. 11 and 12 that with the advancement of the longwall face, both the height of the water-conducting fractured zone and the surface subsidence show a trend of increasing first and then stabilizing. When the height of water-conducting fractured zone is stable, the advancing distance of longwall face shall not exceed the buried depth. While the surface subsidence tends to be stable, the advancing distance is greater than the burial depth. When the height of the water-conducting fractured zone reaches the maximum, the advancing distance of longwall face is 0.4*H* ahead of surface critical mining.

As shown in Table 2, when the height of water-conducting fractured zone remains unchanged at first, the longwall face advances with the buried depth 50 m, 100 m, 220 m and 300 m are *H*, *H*, 0.82*H* and 0.9*H* respectively. When the maximum surface subsidence coefficient is stable first, the advancing distances of 50 m, 100 m, 220 m and 300 m buried depth longwall face are 1.4*H*, 1.4*H*, 1.23*H* and 1.25*H* respectively. As described above, critical advance of longwall face is 0.8*H*–*H* for the maximum height of water-conducting fractured zone, while critical advance of longwall face is 1.2*H*–1.4*H* for the maximum surface subsidence coefficient. Reference^43^ defined the maximum height of water-conducting fractured zone as the overburden critical failure. Combined with the above analysis and related references^44–46^, it is given that the critical advance of longwall face is 0.5*H*–*H* for the overburden critical failure, while the critical advance of longwall face is 1.2*H*–1.4*H* for the surface critical mining under the condition of high-intensity mining. The research results lay a certain foundation for the prediction and control of high-intensity mining damage.

**Figure 13:**
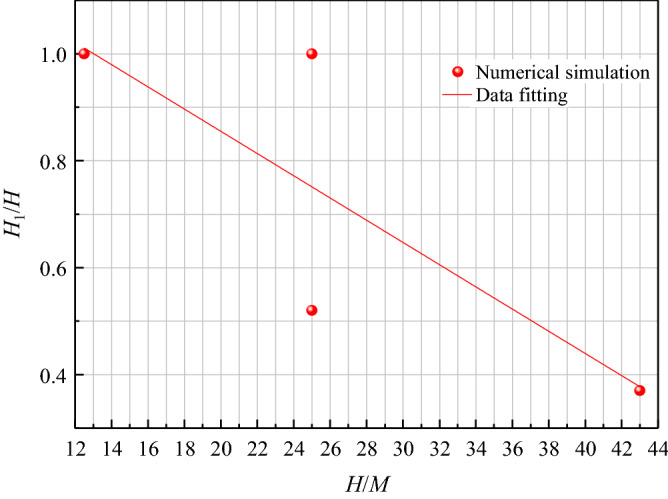
Relationship between *H*/*M* and *H*_1_/*H*.

**Figure 14:**
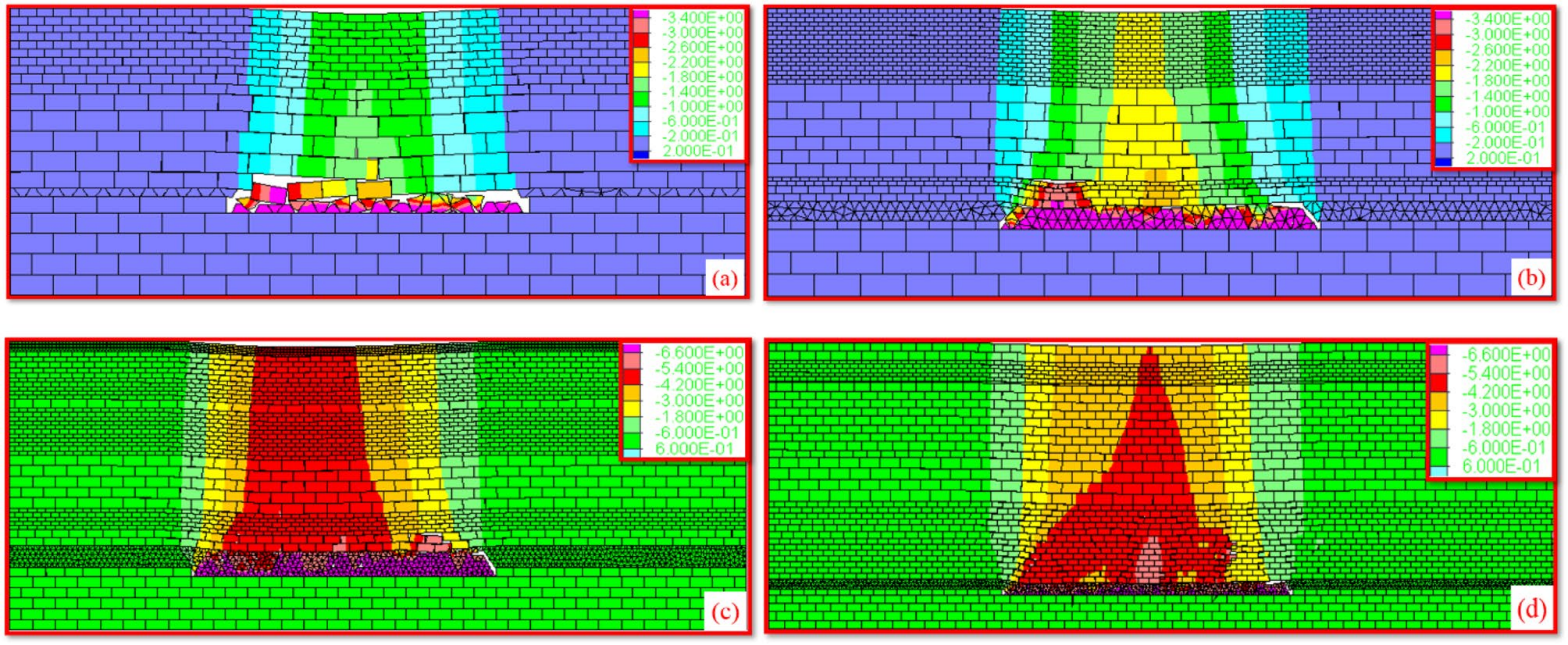
Displacement nephogram of overburden subsidence. (**a**) Subsidence coefficient 0.36. (**b**) Subsidence coefficient 0.47. (**c**) Subsidence coefficient 0.56. (**d**) Subsidence coefficient 0.70.

It can be seen from Figs. 13 and 14 that the larger *H*/*M* is, the smaller *H*_1_/*H* is, and the less likely the surface is to produce discontinuous deformation, while the *H*/*M* has little relationship with the surface subsidence coefficient. For example, the subsidence coefficient of buried depth 50 m (*H*/*M* 12.5, thickness of loose layer 16 m) is 0.36, while the subsidence coefficient of buried depth 100 m (*H*/*M* 25, thickness of loose layer 34 m) is 0.47. This is because the thickness of 100 m loose layer is larger, and the anti-deformation ability of loose layer itself is weak. Once the lower bedrock is broken, it will soon transmit to the surface, resulting in the increase of surface subsidence coefficient; For example, the subsidence coefficient of buried depth 220 m (*H*/*M* 25, mining thickness 8.8 m, thickness of loose layer 9 m) is 0.56, and that of buried depth 300 m (*H*/*M* 43, mining thickness 7 m, thickness of loose layer 20 m) is 0.70, which is also caused by the larger thickness of loose layer. Meanwhile, it can be seen that the sensitivity of the loose layer thickness to the surface subsidence coefficient is greater than that of the mining thickness to the surface subsidence coefficient when the small difference in mining thickness (Δ*M* ≤ 1.8 m), which is consistent with the quantitative study results on the main influencing factors of mining subsidence in reference^47^.

When the buried depth is 50 m and 100 m, and the overburden critical failure *H*_1_/*H* is 1, which indicates that the water-conducting fractured zone is connected with the surface, forming two zones. This is because the coal seam is shallow buried and the bedrock is thin. The mining of 4 m thick coal seam can not form a structure in the overlying rock, and the roof bedrock is cut down, which causes the mining damage to transmit to the surface in a very short time. For example, the mining height of 1203 longwall face in Daliuta coal mine is 4 m, the average buried depth is 60 m, the bedrock thickness is 42 m. There is no bending subsidence zone, resulting in the water gushing and sand breaking accidents^48^. Therefore, the existence of bending subsidence zone has a great impact on the degree of mining damage, so it is very important to build a high-intensity mining damage evaluation system based on bending subsidence zone.

## Surface damage assessment method

According to the field measurement, it is found that the general permanent ground fissures are located above the mining boundary, and the development depth of ground fissures is the largest, which is difficult to close once formed. Since the surface is subcritical mining and the subsidence is very small at the maximum development depth of ground fissure, it is assumed that the maximum development depth of ground fissure is near the mining boundary and the surface subsidence is zero.

Numerical simulation results show that mining damage is a spatiotemporal dynamic evolution process, and the development law of mining cracks is different at different locations and time nodes. Only taking treatment measures through the distribution area of mining damage can not comprehensively and scientifically guide the field work, but also consider the height of water-conducting fractured zone, mining damage types and other factors. The direct form of mining damage is the generation and expansion of mining cracks. The degree of connection between water-conducting fractured zone and ground fissures can reflect the level of mining damage and the degree of impact on ecological environment.

**Figure 15:**
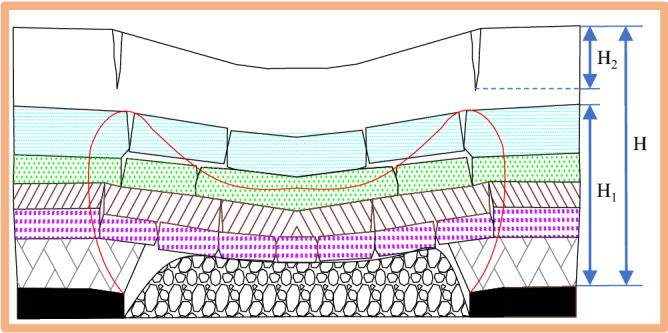
Surface damage assessment diagram.

**Figure 16:**
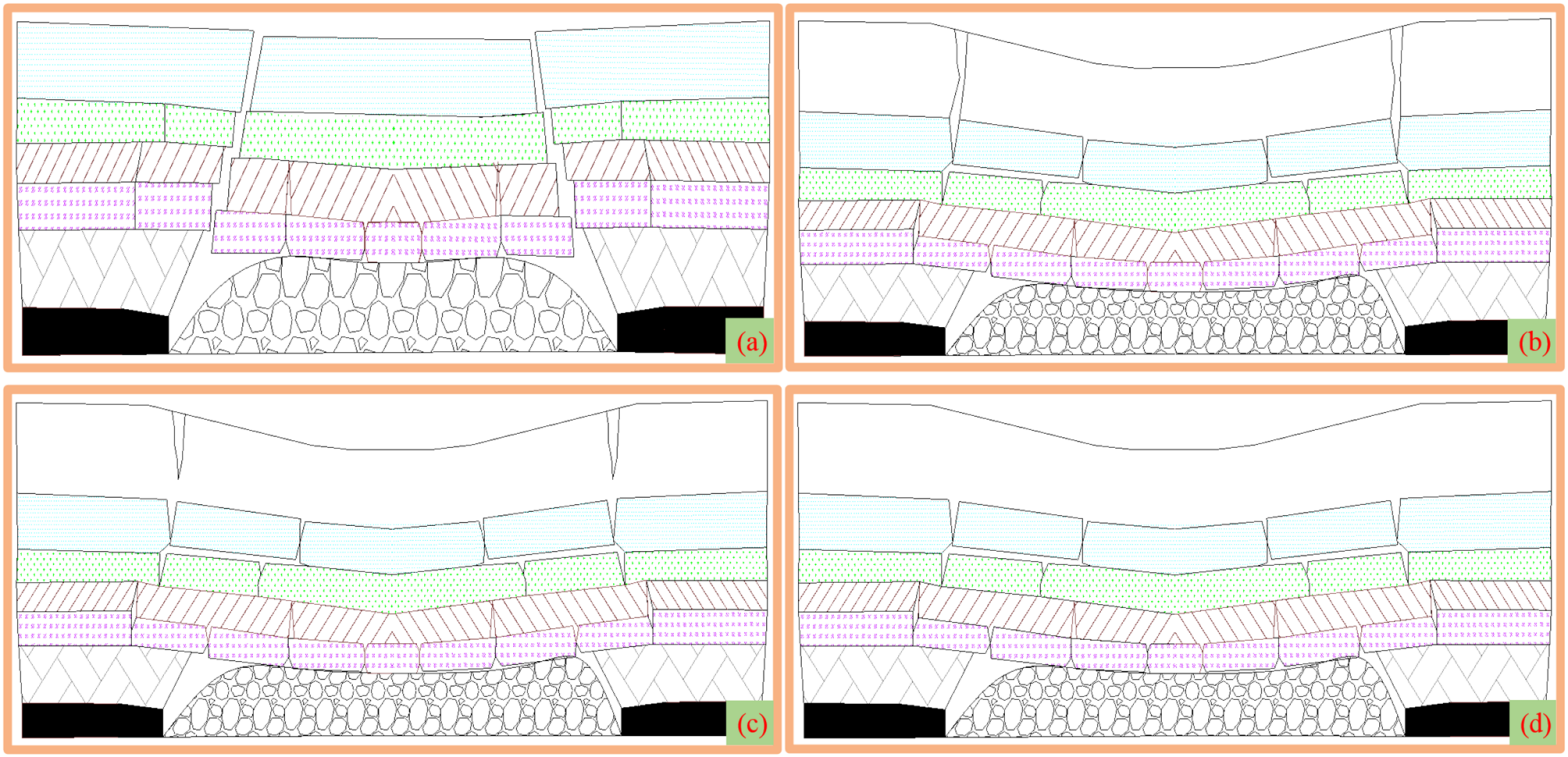
Damage assessment types of high-intensity mining. (**a**) *H*_1_ > *H*. (**b**) *H*_1_ + *H*_2_ = *H*. (**c**) *H*_1_ + *H*_2_ < *H*. (**d**) *H*_2_ = 0.

Therefore, based on the buried depth (*H*), height of water-conducting fractured zone (*H*_1_) and the development depth of ground fissures (*H*_2_) (Fig. 15), according to whether there is bending subsidence zone and the water-conducting fractured zone is connected with ground fissures, the high-intensity damage development is divided into four types (Fig. 16). Considering the types and distribution area of ground fissures, ecological environment vulnerability and other factors, the damage mechanism and control method of high-intensity mining (Table 3) are given to scientifically guide the field practice and ecological restoration.

**Table 3:** Surface damage assessment system and control method of high-intensity mining^49^.

Control methods	Ground fissure area	Types of ground fissures	Damage types	Damage characteristics	Ecological environment vulnerability	Damage grade	Control methods
(b) (c)	A	Stepped type	*H*_1_ > *H*	There is no bending subsidence zone, and the water-conducting fractured zone is connected with the surface	Extremely fragile, highly fragile	I_1_	(a)
					Moderately fragile, lightly fragile	I_2_	(a) (b)
	B						
					Not fragile	I_3_	(b) (c)
(c) (e)	A	Stepped type	*H*_1_ + *H*_2_ = *H*	There is a bending subsidence zone, and the water-conducting fractured zone is connected with the ground fissures	Extremely fragile, highly fragile	II_1_	(a) (b)
					Moderately fragile, lightly fragile	II_2_	(b) (c)
	B						
					Not fragile	II_3_	(c)
(c) (e)	A	Tensile type	*H*_1_ + *H*_2_ < *H*	There is a bending subsidence zone, and the water-conducting fractured zone is not connected with the ground fissures	Extremely fragile, highly fragile	III_1_	(b) (c)
					Moderately fragile, lightly fragile	III_2_	(c)
(e) (f)	B						
					Not fragile	III_3_	(c) (d)
(f)	A	No ground fissure	*H*_2_ = 0	There is bending subsidence zone and no ground fissure	Extremely fragile, highly fragile	IV_1_	(c)
					Moderately fragile, lightly fragile	IV_2_	(c) (d)
	B						
					Not fragile	IV_3_	(d)

A: Boundary permanent fissures zone; B: Middle dynamic fissures zone; (a) No mining; (b) Fully filled mining; (c) Partial filling, coal pillar retaining, height limited mining and grouting in key strata; (d) Direct mining; (e) Artificial landfill; (f) Self-repair.

It can be seen from Table 3 that the surface damage assessment method divides the damage grades into four categories according to the distribution characteristics of overburden fracture field, and the damage degree decreases in turn. Combined with the classification standard of ecological environment sensitivity in the mining area, it is divided into twelve sub-categories: I_1_, I_2_, I_3_, II_1_, II_2_, II_3_, III_1_, III_2_, III_3_, IV_1_, IV_2_ and IV_3_. Based on the classification of grades, it is proposed that related technical measures are scientifically applied to specific production practices and ecological environmental protection.

## Field application

Taking the 12,401 longwall face of Shangwan coal mine as the field application, the height of caving zone is 33 m, the height of fracture zone is 124 m. Statistics show that 92% of the ground fissures are within 4 m in depth. Through the field detection of high-density electrical method and ground penetrating radar, the water-conducting fractured zone is not connected with the ground fissures, and the ground fissures only develops in the aeolian sand area. Based on the damage development mechanism of high-intensity mining, longwall face rapid advancing method, local grouting reinforcement overburden method and zoning treatment ground fissures method are used to realize the full cycle mining damage control from the source (longwall face), the development process (overburden rock) and the end treatment (surface).

**Figure 17:**
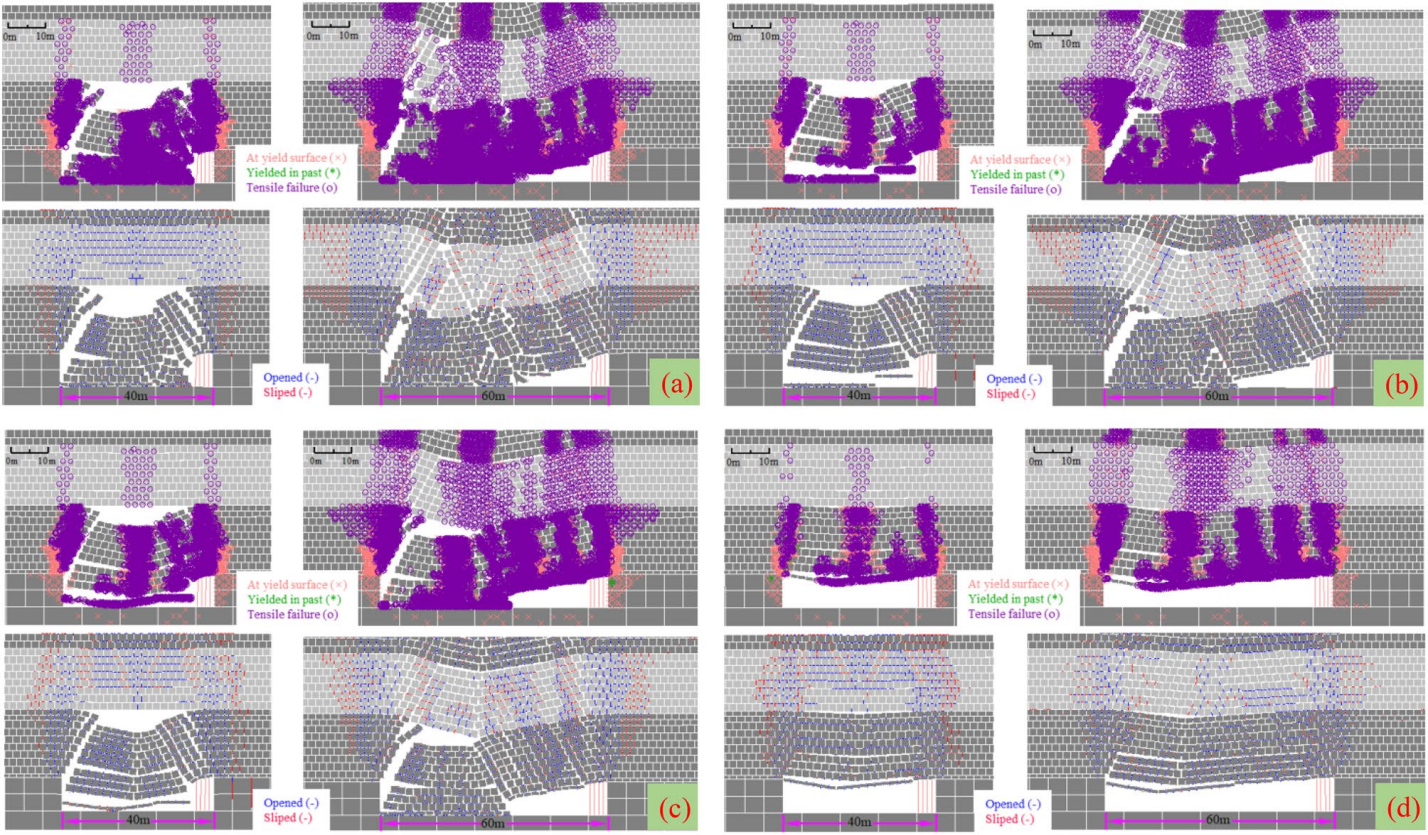
Mining damage degree with different advancing speed. (**a**) 5 m/d. (**b**) 10 m/d. (**c**) 15 m/d. (**d**) 20 m/d.

**Figure 18:**
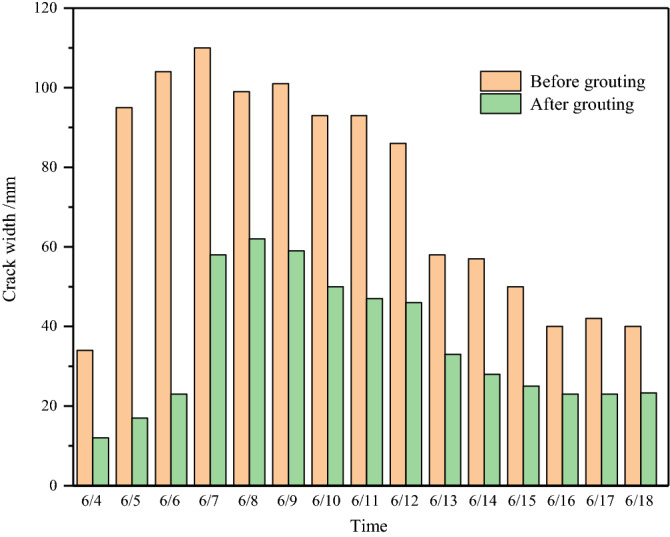
Ground fissure treatment effect.

**Figure 19:**
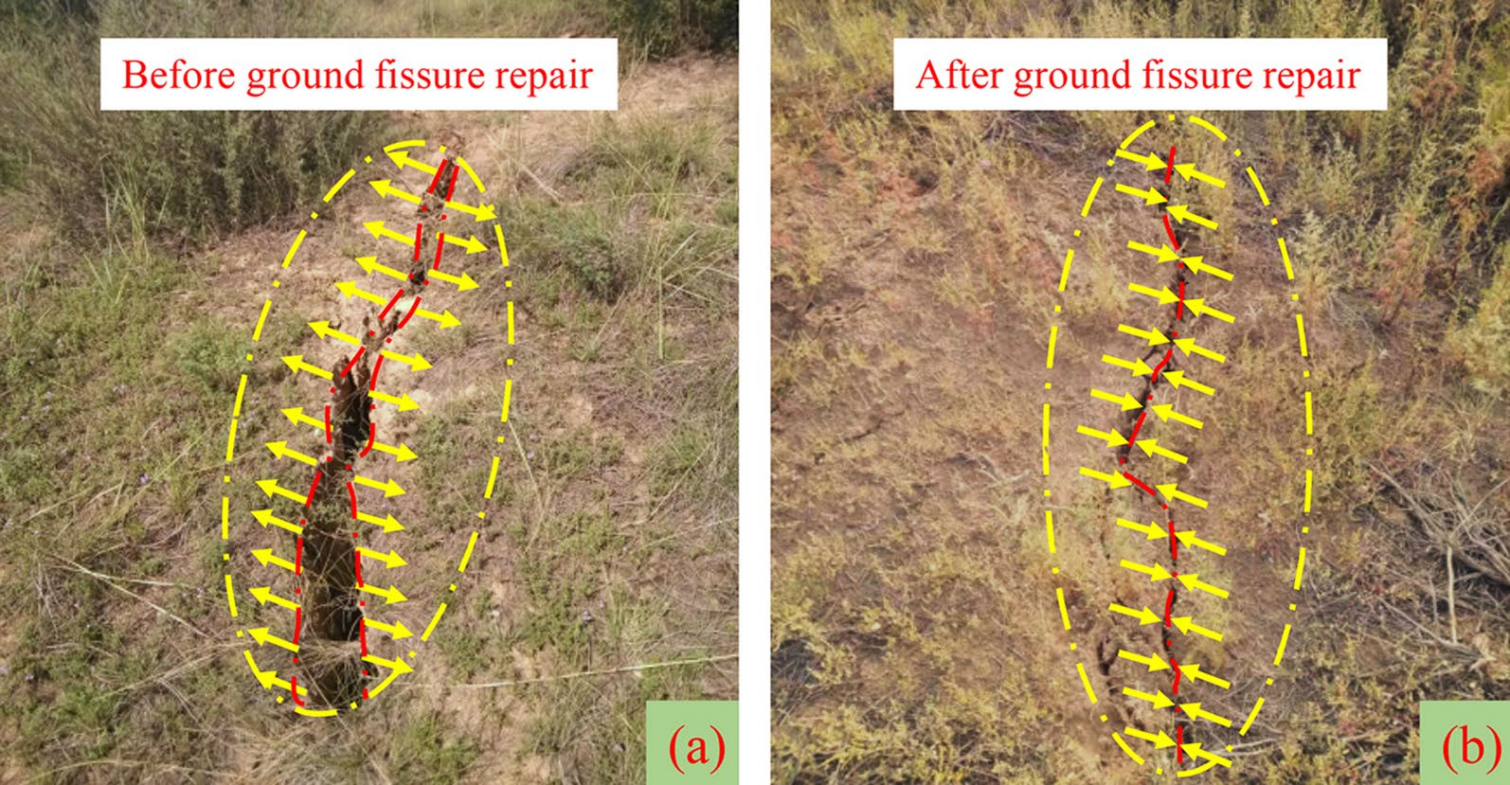
Ground fissure repair effect. (**a**) Before ground fissure repair. (**b**) After ground fissure repair.

1. Longwall face rapid advancing method. In the early stage of mining, the average advancing speed is less than 4 m/d, and the overburden and surface damage are serious. In the later stage, the average advancing speed reaches 13.7 m/d. The overburden pressure relief is insufficient, and the main roof weighting step increases. The damage development shows a certain lag in time and space, and the damage degree decreases. As shown in Fig. 17, the mining damage degree is verified with different advancing speeds based on the field simulation.
2. Local grouting reinforcement overburden method. The local grouting reinforcement method of overburden is realized by grouting in the caving zone and the separated fracture zone. The grouting in the caving zone can maintain the main roof stability, reduce the roof subsidence and the degree of rock movement and deformation above the caving zone. Grouting in the separated fracture zone can control the subsidence of the key strata, further strengthen the key strata, limit the mining damage in the overburden, reduce the overburden damage height, promote the appearance of "three zones" mode, and weaken the surface damage degree. Figure 18 shows that grouting promotes the development of cracks more gently, and the width of cracks is significantly reduced, which can effectively control mining damage.
3. Zoning treatment ground fissures method. For the permanent ground fissures in the mining boundary, the control method of source reduction damage and reinforcement of the key strata is adopted. For the dynamic ground fissures in the middle, the fissures that can be closed are allowed to self-repair. For the fissures that cannot be closed, the method of artificial landfill is adopted. Figure 19 shows the comparison before and after the treatment of mining induced ground fissures.

## Conclusions

1. Surface damage process undergone five stages. The breakage and migration of key stratum is the key reason to cause surface damage. Under the critical extraction condition, the cracks above the goaf are divided into five areas, and a deviant subsidence basin is formed on the surface.
2. The overburden reaches critical failure ahead of surface critical mining. In the case of small difference in mining thickness (Δ*M* ≤ 1.8 m), the sensitivity of loose layer thickness to surface subsidence coefficient is greater than that of mining thickness to surface subsidence coefficient.
3. Based on the bending subsidence zone and the connection degree of mining-induced cracks in the rock and soil layers, surface damage evaluation method and other related control measures are proposed and successfully applied in the 12,401 longwall face of Shangwan coal mine.

## Data Availability

The data used to support the findings of this research are included within the paper.
